# Scoping review of rehabilitation care models for post COVID-19 condition

**DOI:** 10.2471/BLT.22.288105

**Published:** 2022-10-03

**Authors:** Simon Décary, Wouter De Groote, Chiara Arienti, Carlotte Kiekens, Paolo Boldrini, Stefano Giuseppe Lazzarini, Michèle Dugas, Théo Stefan, Léa Langlois, Frédérique Daigle, Florian Naye, Annie LeBlanc, Stefano Negrini

**Affiliations:** a Faculty of Medicine and Health Sciences, Research Centre of the CHUS, University of Sherbrooke, 3001, 12e Avenue Nord, Sherbrooke, Quebec, J1H 5N4, Canada.; b Department for Noncommunicable Diseases, World Health Organization, Geneva, Switzerland.; c IRCCS Fondazione Don Gnocchi, Milan, Italy.; d IRCCS MultiMedica, Milan, Italy.; e Italian Society of Physical and Rehabilitation Medicine, Roma, Italy.; f VITAM Research Center on Sustainable Health, Quebec Integrated University Health and Social Services Center, Québec, Canada.; g Department of Biomedical, Surgical and Dental Sciences, University “La Statale”, Milan, Italy.

## Abstract

**Objective:**

To systematically map the current evidence about the characteristics of health systems, providers and patients to design rehabilitation care for post coronavirus disease 2019 (COVID-19) condition.

**Methods:**

We conducted a scoping review by searching the databases: MEDLINE®, Embase®, Web of Science, Cochrane COVID-19 Registry and Cochrane Central Register of Controlled Trials, from inception to 22 April 2022. The search strategy included terms related to (i) post COVID-19 condition and other currently known terminologies; (ii) care models and pathways; and (iii) rehabilitation. We applied no language or study design restrictions. Two pairs of researchers independently screened title, abstracts and full-text articles and extracted data. We charted the evidence according to five topics: (i) care model components and functions; (ii) safe delivery of rehabilitation; (iii) referral principles; (iv) service delivery settings; and (v) health-care professionals.

**Findings:**

We screened 13 753 titles and abstracts, read 154 full-text articles, and included 37 articles. The current evidence is conceptual and expert based. Care model components included multidisciplinary teams, continuity or coordination of care, people-centred care and shared decision-making between clinicians and patients. Care model functions included standardized symptoms assessment, telehealth and virtual care and follow-up system. Rehabilitation services were integrated at all levels of a health system from primary care to tertiary hospital-based care. Health-care workers delivering services within a multidisciplinary team included mostly physiotherapists, occupational therapists and psychologists.

**Conclusion:**

Key policy messages include implementing a multilevel and multiprofessional model; leveraging country health systems’ strengths and learning from other conditions; financing rehabilitation research providing standardized outcomes; and guidance to increase patient safety.

## Résumé

**Objectif:**

Recenser systématiquement les données probantes actuelles concernant les caractéristiques des systèmes de santé, prestataires et patients afin de définir un modèle de réadaptation pour les affections consécutives à la maladie à coronavirus 2019 (COVID-19).

**Méthodes:**

Nous avons mené une analyse exploratoire en étudiant plusieurs bases de données: MEDLINE®, Embase®, Web of Science, Registre Cochrane des études sur la COVID‐19 et Registre central Cochrane des essais contrôlés, depuis le début et jusqu'au 22 avril 2022. La stratégie de recherche consistait à inclure des termes en lien avec (i) les affections post-COVID-19 et d'autres terminologies connues à ce jour; (ii) les modèles et parcours de soins; et enfin, (iii) la réadaptation. Nous n'avons appliqué aucune restriction de langue ou de format d'étude. Deux binômes de chercheurs ont passé au crible les titres, résumés et articles complets, indépendamment l'un de l'autre, pour ensuite en extraire des informations. Nous avons établi une grille de données probantes autour de cinq thèmes: (i) éléments et fonctions des modèles de soins; (ii) fourniture de services de réadaptation sûrs; (iii) principes de référence; (iv) lieux de prestation des services; et enfin, (v) professionnels de la santé.

**Résultats:**

Nous avons examiné 13 753 titres et résumés, lu 154 articles complets et inclus 37 d'entre eux. Les données probantes actuelles reposent sur des concepts et expertises. Les éléments des modèles de soins impliquent des équipes multidisciplinaires, une continuité ou une coordination des soins, des prestations axées sur l'humain et une prise de décision partagée entre médecins et patients. Parmi les fonctions des modèles de soins figurent l'évaluation standardisée des symptômes, la télémédecine et les soins virtuels, ainsi que le mécanisme de suivi. Les services de réadaptation sont présents à tous les niveaux du système de santé, des soins primaires aux soins tertiaires en milieu hospitalier. Les équipes multidisciplinaires en charge de la réadaptation sont principalement composées de kinésithérapeutes, d'ergothérapeutes et de psychologues.

**Conclusion:**

Cette démarche véhicule d'importants messages politiques: la mise en œuvre d'un modèle comprenant plusieurs niveaux et professions; une valorisation des atouts du système sanitaire en vigueur dans le pays, ainsi qu'une pratique qui s'inspire d'autres affections; le financement de recherches sur la réadaptation afin de parvenir à des résultats standardisés; et enfin, des conseils pour améliorer la sécurité des patients.

## Resumen

**Objetivo:**

Esbozar de manera sistemática la evidencia actual sobre las características de los sistemas sanitarios, los proveedores y los pacientes con el fin de diseñar la atención de rehabilitación para la afección posterior a la enfermedad por coronavirus de 2019 (COVID-19).

**Métodos:**

Se realizó una revisión exploratoria mediante búsquedas en las bases de datos: MEDLINE®, Embase®, Web of Science, Registro Cochrane COVID-19 y Registro Cochrane Central de Ensayos Controlados, desde su inicio hasta el 22 de abril de 2022. La estrategia de búsqueda incluyó términos relacionados con (i) afección posterior a la COVID-19 y otras terminologías que se conocen en la actualidad; (ii) modelos y vías de atención; y (iii) rehabilitación. No se aplicó ninguna restricción de idioma o de diseño del estudio. Dos pares de investigadores revisaron de forma independiente el título, los resúmenes y los artículos de texto completo y extrajeron los datos. Se clasificó la evidencia de acuerdo con cinco temas: (i) componentes y funciones del modelo de atención; (ii) prestación segura de la rehabilitación; (iii) principios de derivación; (iv) entornos de prestación de servicios; y (v) profesionales sanitarios.

**Resultados:**

Se revisaron 13 753 títulos y resúmenes, se leyeron 154 artículos de texto completo y se incluyeron 37 artículos. La evidencia actual es conceptual y de especialistas. Los componentes de los modelos de atención incluyeron equipos multidisciplinarios, continuidad o coordinación de la atención, atención orientada a las personas y toma de decisiones compartida entre los médicos y los pacientes. Las funciones del modelo de atención incluyeron la evaluación estandarizada de los síntomas, la telemedicina y el sistema de atención y seguimiento virtual. Los servicios de rehabilitación se integraron en todos los niveles de un sistema sanitario, desde la atención primaria hasta la atención hospitalaria terciaria. El personal sanitario que prestaba servicios dentro de un equipo multidisciplinar incluía en su mayoría fisioterapeutas, terapeutas ocupacionales y psicólogos.

**Conclusión:**

Los mensajes de políticas más importantes incluyen la aplicación de un modelo multinivel y multiprofesional; el aprovechamiento de las fortalezas del sistema sanitario del país y el aprendizaje de otras afecciones; la financiación de la investigación en materia de rehabilitación que proporcione resultados estandarizados; y la orientación para aumentar la seguridad de los pacientes.

## ملخص

الغرض عمل ربط منهجي للأدلة الحالية المتعلقة بخصائص النُظم الصحية، وموفري الخدمات والمرضى من أجل تصميم الرعاية التأهيلية اللازمة للحالات الصحية اللاحقة لمرض فيروس كورونا (كوفيد 19) في عام 2019.

الطريقة أجرينا مراجعة للنطاق من خلال البحث في قواعد البيانات التالية: ®MEDLINE، و®Embase، وWeb of Science (شبكة العلوم)، وسجل كوكرين الخاص بكوفيد 19 وسجل كوكرين المركزي للتجارب الخاضعة للتحكم، من بداياتها وحتى يوم 22 أبريل/نيسان 2022. تضمنت استراتيجية البحث مصطلحات ترتبط بمواضيع تشمل: (1) الحالات الصحية اللاحقة لكوفيد 19 والمصطلحات الأخرى المعروفة في الوقت الحالي؛ و(2) نماذج ومسارات الرعاية؛ و(3) إعادة التأهيل. لم نضع أي قيود تتعلق باللغة أو تصميم الدراسة. قام زوجان من الباحثين بشكل مستقل بفرز العناوين والملخصات والنصوص الكاملة للمقالات والبيانات المستخلصة منها. لقد وضعنا مخططًا للأدلة على أساس خمسة مواضيع: (1) مكونات ووظائف نموذج الرعاية؛ و(2) التقديم الآمن لخدمات إعادة التأهيل؛ و(3) مبادئ الإحالة؛ و(4) أماكن تقديم الخدمات؛ و(5) مهنيو الرعاية الصحية.

النتائج نجحنا في فرز 13 753 عنوانًا وملخصًا، وأكملنا قراءة 154 مقالة نصية كاملة، وتضمين 37 مقالة. الأدلة الحالية تعتمد على كلٍ من المفاهيم ومساهمات الخبراء. شملت مكونات نماذج الرعاية فرق تضم تخصصات متعددة، واستمرارية الرعاية أو تنسيق جهودها، والرعاية التي تركز على مصالح الأشخاص، وتقاسم اتخاذ القرارات بين الأطباء السريريين والمرضى. شملت وظائف نموذج الرعاية تقييم الأعراض الموحدة، وتقديم خدمات الرعاية الافتراضية وعن بُعد ونظام المتابعة. تم إدماج خدمات إعادة التأهيل في جميع مستويات النظام الصحي بدءًا من الرعاية الأولية وصولاً إلى الرعاية المتخصصة داخل المستشفيات. تتألف القوة العاملة في مجال تقديم خدمات الرعاية الصحية ضمن فريق عمل متعدد التخصصات في أغلب الأحيان من الاختصاصيين في العلاج الطبيعي وعلم النفس والمعالجين المهنيين.

الاستنتاج تتضمن الرسائل الأساسية المرجوة من السياسات تطبيق نموذج متعدد المستويات يضم مهارات مهنية متنوعة؛ والاستفادة من نقاط القوة الكامنة في النظام الصحي للبلاد والتعلم من الحالات الصحية الأخرى؛ وتمويل أبحاث إعادة التأهيل التي تؤدي إلى نتائج موحدة؛ وتوفير التوجيه اللازم لرفع مستوى سلامة المرضى.

## 摘要

**目的:**

旨在系统地规划有关卫生系统、护理提供者和患者特征的当前证据,设计新型冠状病毒肺炎 (COVID-19) 大流行后时代的康复护理模型。

**方法:**

我们通过搜索MEDLINE®、Embase®、Web of Science、Cochrane COVID-19 注册和 Cochrane 对照试验中央注册中心等数据库从创立日至 2022 年 4 月 22 日期间的数据,进行了范围审查。搜索策略是对与以下内容相关的术语进行搜索:(i) COVID-19 后时代和其他当前已知术语;(ii) 护理模型和途径;(iii) 康复。我们没有限制语言或研究设计。两组研究人员独立筛选标题、摘要和论文全文并提取相关数据。我们根据五个主题规划了相关证据:(I) 护理模型组成部分和功能;(ii) 安全提供康复服务;(iii) 转介原则;(iv) 服务交付设置;(v) 医疗保健专业人员。

**结果:**

我们筛选了 13,753 篇论文的标题和摘要,阅读了 154 篇论文全文,并收录了 37 篇论文。当前证据根据概念定义和专家意见进行规划。护理模型的组成部分包括多学科团队、护理的持续性或协调性、以人为本的护理以及医患共同决策。护理模型功能包括标准化症状评估、远程医疗、虚拟护理和随访系统。康复服务被整合到卫生系统的各个层面(从初级保健到三级医院护理)。在多学科团队中提供服务的医疗保健工作者主要包括物理治疗师、职业治疗师和心理医生。

**结论:**

关键政策措施包括实施多层次和多专业模型;利用国家卫生系统的优势并从其他情况中吸取经验教训;资助提供标准化结果的康复研究;以及提供加强患者安全保障方面的指导。

## Резюме

**Цель:**

Систематически отобразить текущие данные о характеристиках систем здравоохранения, провайдерах и пациентах для разработки реабилитационной помощи в условиях постковидного синдрома 2019-(COVID-19).

**Методы:**

Авторы провели обзорный анализ путем поиска в базах данных: MEDLINE®, Embase®, Web of Science, Кокрановском реестре COVID-19 и Кокрановском центральном реестре контролируемых исследований – с момента создания до 22 апреля 2022 г. Стратегия поиска включала термины, связанные с (i) постковидным синдромом и другими, известными в настоящее время терминами, (ii) моделями и способами оказания медицинской помощи и (iii) реабилитацией. Авторы не применяли ограничений по языку или дизайну исследования. Две независимые группы исследователей тщательно проверили заголовки, выдержки и полнотекстовые статьи, а затем извлекли данные. Авторы распределили доказательства по пяти темам: (i) компоненты и функции модели оказания медицинской помощи, (ii) безопасное предоставление реабилитации, (iii) принципы в отношении направления к специалисту, (iv) условия оказания услуг и (v) медицинские работники.

**Результаты:**

Авторы просмотрели 13 753 заголовка и выдержки, прочитали 154 полнотекстовые статьи и проанализировали 37 статей. Имеющиеся в настоящее время данные носят концептуальный характер и основаны на экспертных оценках. Компоненты моделей оказания медицинской помощи включали многопрофильные группы, непрерывность или координацию помощи, социально ориентированную помощь, а также совместное принятие решений между врачами и пациентами. Функции модели оказания медицинской помощи включали: стандартизированную оценку симптомов, телемедицину, а также виртуальную систему оказания помощи и последующего наблюдения. Реабилитационные услуги были интегрированы на всех уровнях системы здравоохранения: от первичной медико-санитарной помощи до специализированной больничной помощи. Медицинские работники, оказывающие услуги в рамках многопрофильной группы, включали в основном физиотерапевтов, эрготерапевтов и психологов.

**Вывод:**

Ключевые моменты политики включают в себя внедрение многоуровневой и многопрофессиональной модели, использование сильных сторон национальной системы здравоохранения и изучение других условий, финансирование реабилитационных исследований, обеспечивающих стандартизированные результаты, и рекомендации по повышению безопасности пациентов.

## Introduction

People living with post coronavirus disease 2019 (COVID-19) condition, first described as long COVID, need recognition and rehabilitation.^1^ The World Health Organization (WHO) has created a definition for post COVID-19 condition, that is, *“*history of probable or confirmed [severe acute respiratory syndrome coronavirus 2] infection, usually 3 months from the onset, with symptoms that last for at least 2 months and cannot be explained by an alternative diagnosis. Common symptoms include, but are not limited to, fatigue, shortness of breath and cognitive dysfunction, and generally have an impact on everyday functioning. Symptoms may be new onset following initial recovery from an acute COVID-19 episode or persist from the initial illness. Symptoms may also fluctuate or relapse over time.”^2^

A survey of 3762 people with post COVID-19 condition identified that three out of four were still experiencing fatigue, post-exertional symptom exacerbation and cognitive dysfunction after 6 months, and half were unable to fully return to work.^3^ Convergent with prospective cohorts, between a third to three quarters of hospitalized and community patients had not recovered at 6 and 12 months and a fifth of patients described persistent functional limitations.^4^^–^^8^ The multisystemic, fluctuating or episodic and relapsing nature of post COVID-19 condition^9^^,^^10^ is confirmed by a systematic review of 47 910 patients.^11^

A systematic review reporting on 886 388 COVID-19 patients estimated a pooled prevalence of post COVID-19 condition at 43% (95% confidence interval: 35–63).^12^ As of April 2022, the authors estimated that about 100 million people had or are still living with post COVID-19 condition worldwide. Disabling symptoms affect quality of life, return to work or school, finances and ability to care for self and their families.^13^^–^^15^ The scale of this international public health issue could overwhelm health-care capacity, particularly in low- and middle-income countries.

The multisystemic characteristics of the post COVID-19 condition and its high prevalence cause issues for health systems management, with the need to identify appropriate care models. Innovative post-COVID clinics highlighted the need for continuity of care and multidisciplinary rehabilitation.^11^^,^^16^^–^^18^ Shortcomings are appearing such as long waiting lists, difficulties training clinicians, delivery of safe rehabilitation, barriers to access for patients with fatigue, absence of integrated rehabilitation and funding sustainability.^19^

The objective of this scoping review is to systematically map the evidence about health system, providers and patients’ characteristics to guide decision-makers in designing sustainable rehabilitation care models for post COVID-19 condition.

## Methods

The protocol follows the Joanna Briggs Institute guidelines for scoping reviews^20^ and the framework from Arksey & O’Malley^21^ and Levac et al.^22^ We report our review according to the Preferred Reporting Items for Systematic Reviews and Meta-Analyses Extension for Scoping Reviews guidelines.^23^

### Concept and research questions

Rehabilitation is defined as interventions for people with limitations in daily physical, mental and social functioning and aims to help them achieve their optimal level of functioning in their environment.^24^ We define a rehabilitation care model as the organizational structure required to deliver rehabilitation interventions within a health system. Care models rely on multiple possible active components required to support the delivery of services. Components also benefit from functions to support the operationalization of the different components that constitute a care model.

Our research question was: what is known about health system, providers and patients’ characteristics to design rehabilitation care models for post COVID-19 condition? We explored the two broad concepts of care models and rehabilitation for the specific population of post COVID-19 condition.

To answer our research question, we defined five topics relevant to decision-makers (Box 1), which we based on a previous living systematic review on rehabilitation interventions for post COVID-19 condition^26^^,^^27^ and a rapid systematic review on care models for post COVID-19 condition.^28^

Box 1 **Data charting framework to classify concepts on rehabilitation care models for post COVID-19 condition**

To answer our research question “What is known about health system, providers and patients’ characteristics to design rehabilitation care models for post COVID-19 condition?” we defined five topics relevant to decision-makers.

**Topic 1: Components and functions of rehabilitation care models**

Research question: (i) what are the core components and functions of rehabilitation care models?

We define a care model as the organizational structure required to deliver health services and interventions within a health system. We propose that care models rely on multiple possible active components required to support the delivery of services. Components also benefit from functions as mechanisms or tools to support the operationalization of the different components that constitute a care model. Complete definitions are available in the data repository.^25^

We searched for description of the following components: patient-centred care and shared decision-making, patient education, guided self-management (supported recovery), integrated care, multidisciplinary teams, shared care, continuity or coordination of care, case management, patient navigators, one-stop-shop clinics, asynchronous care, evidence-based care, community of practice, quality improvement, patient-reported outcome measures evaluation, training for health-care professionals or research partnership.

We searched for description of the following functions: decision support for health care professionals, clinical information system, triage system, standardized symptoms assessment, social determinants assessment, referral system, follow-up system, patient support groups, home-based care, telehealth/virtual care.

**Topic 2: Safe delivery of rehabilitation**

Research question: (ii) to ensure safe rehabilitation, which COVID-19-related symptoms and conditions and/or complications (e.g. myocarditis, arrythmia, pulmonary emboli, severe desaturation) need further investigation and/or treatment and management before referral to general or specific rehabilitation interventions?

Safe delivery of rehabilitation includes the identification of conditions or symptom clusters that need management before referral for rehabilitation.

**Topic 3: Rehabilitation referral principles**

Research questions: (iii) who should be referred for rehabilitation services and what would be relevant criteria for referral into rehabilitation?; and (iv) what is the proposed timing for referral into rehabilitation?

We define criteria for referral as the identification of relevant patient-level characteristics for referral into rehabilitation, regardless of the referral type. For example, criteria based on severity of symptoms, initial disease severity, overlap of symptom clusters, risk factors for development of persistent limitations in functioning, limitations in functioning assessed with a scale (e.g. Post COVID-19 functional status scale), abnormal clinical findings, outcome measures (impairment), patient-reported outcome measures (e.g. health-related quality of life), return to work or amenability to rehabilitation.

We define timing for referral as the decision about when the optimal timing is for referral into rehabilitation, regardless of the referral type.

**Topic 4: Rehabilitation services delivery setting**

Research questions: (v) what rehabilitation service delivery mode could be used for the provision of post COVID-19 condition rehabilitation services?; (vi) what type of rehabilitation care organization is needed?; (vii) what support mechanisms to facilitate delivery of rehabilitation services should be put in place?; (viii) should service organization be integrated or parallel with regards to existing health care system?; (ix) what would be the ideal length of programme?

Examples of rehabilitation delivery mode are face-to-face, virtual or group delivery; examples of rehabilitation care organization are hospital-based, community based or in primary care; examples of support mechanisms are follow-up system or monitoring.

**Topic 5. Health-care professionals providing rehabilitation interventions**

Research questions: (x) what common health-care professionals are involved in the provision of rehabilitation interventions for post COVID-19 condition?; (xi) what type of competencies and skills (e.g. evidence-based practice, communication, education, monitoring) are required for post COVID-19 condition rehabilitation?; and (xii) what number of years of clinical experience is required to safely work with the post COVID-19 condition population?

We define common health-care professionals as appropriate professions to develop a core team or appropriate staffing of a post COVID-19 condition rehabilitation service.

COVID-19: coronavirus disease 2019.

Topic 1: what are core components and functions of rehabilitation care in people with post COVID-19 condition? This topic relates to rehabilitation components and functions, which are the active organizational structure required to support the delivery of services and their supporting mechanisms. We present definitions used for proposed components and functions in our data repository.^25^

Topic 2: what are conditions for the safe delivery of rehabilitation? This topic relates to the safe delivery of rehabilitation by identifying conditions or symptoms that need management before referral for rehabilitation.

Topic 3: what are the referral principles that need to be considered? This topic relates to identifying relevant patient-level characteristics and criteria and timing for referral into rehabilitation.

Topic 4: in which setting should rehabilitation be provided? This topic relates to describing service delivery setting such as delivery mode, delivery platform, support mechanisms, integration within health system and length of programmes.

Topic 5: what professions need to be involved in the rehabilitation of people with post COVID-19 condition? This topic relates to describing the workforce characteristics required to provide rehabilitation interventions such as common rehabilitation workers, type of competencies and skills, and years of clinical experience.

### Eligibility criteria

We included studies of any design meeting the following criteria: (i) studying adult population with post COVID-19 condition (WHO clinical case definition);^2^ (ii) reporting on any aspects of rehabilitation care models to answer our defined research questions. We included studies describing complete care models (e.g. pathways, frameworks or structured clinics) and their components and functions, regardless of whether the studies included a comparator or not. Studies reporting any system-level outcomes (e.g. cost–effectiveness, access), provider-level outcomes (e.g. satisfaction, confidence in providing care) or patient-level outcomes (e.g. improved functioning, patient-reported outcome measures, return to work) were eligible.

### Search strategy

We systematically searched MEDLINE®, Embase®, Web of Science, Cochrane COVID-19 Registry and Cochrane Central Register of Controlled Trials for studies. An experienced medical information specialist developed and tested the search strategy. The complete search strategy is provided in the data repository.^25^ In short, the strategy included terms related to: (i) post COVID-19 condition and other currently known terminologies (e.g. post-acute sequelae of COVID-19, long COVID, post COVID-19 syndrome); (ii) care models and pathways (e.g. health-care organization); and (iii) rehabilitation. We built the search string using MeSH terms and free-text terms linked with Boolean collectors (AND, OR, NOT) without any language or study design restriction. Our secondary information sources included a manual search of reference lists or related citations, non-peer reviewed materials including book chapters, governmental agency reports and websites, position papers or proceedings of conferences. In line with the scoping review method, our search strategy was not restrictive and identified citations for our broad aim’s main concepts of care models and rehabilitation for post COVID-19 condition.

The search was performed from inception to 24 September 2021 and updated to 22 April 2022. We merged citations from all information sources and we removed duplicates using EndNote version X9 (Clarivate, Philadelphia, United States of America).

### Selection process

Two pairs of reviewers independently screened each title and abstract using the eligibility criteria. They read relevant full-text articles and systematically applied eligibility criteria. Disagreement was settled using a consensus approach between the two reviewers. Discrepancies were resolved by discussion or by a third senior researcher.

### Data extraction and charting

Using the topics and questions presented in Box 1, we developed a data extraction and charting framework to map evidence about health system, providers, and patients’ characteristics of rehabilitation care models for post COVID-19 condition. We categorized the extracted data using the topics and questions presented in Box 1.

### Risk of bias assessment

We planned to conduct an assessment of risk of bias by two reviewers using the Cochrane Risk of Bias tool^29^ on any included randomized controlled trials (RCTs) and nonrandomized controlled trials.

### Data synthesis

We describe characteristics of the included studies (e.g. years, countries, population, type of care model) using simple descriptive statistics (frequency and percentage). We conducted a thematic content analysis centred on the five topics and signalling questions. We present a narrative synthesis of information and created a concept map of identified evidence. 

## Results

### Study selection

**Fig. 1:**
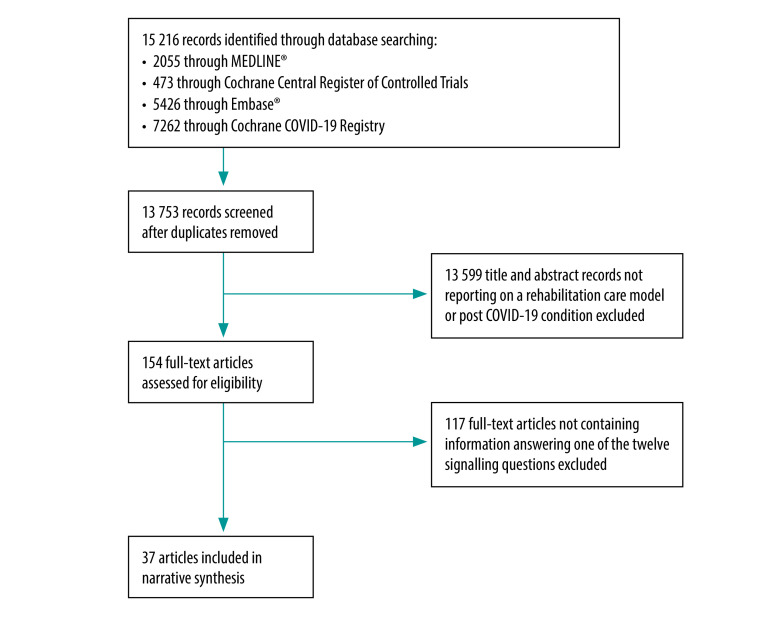
Selection of studies on designing rehabilitation care for post COVID-19 condition

After duplicate removal, we identified 13 753 titles and abstracts (Fig. 1). We read 154 full texts and included 37 articles reporting information related to the five topics and 12 questions on rehabilitation care models.^17^^,^^30^^–^^65^

COVID-19: coronavirus disease 2019.

### Characteristics of articles

Seven out of 37 included articles were published in 2020, 27 were published in 2021 and 3 were published in 2022 (Table 1). Sixteen articles were conducted in the United Kingdom of Great Britain and Northern Ireland. All but one of the studies were conducted in high- and upper-middle-income countries. The number of participants per study ranged from 14 to 1325. No articles reported on children. When described, the settings of rehabilitation care models were outpatient rehabilitation (11 studies),^17^^,^^35^^,^^38^^,^^44^^,^^46^^,^^51^^,^^53^^,^^55^^,^^59^^,^^62^^,^^63^ community-based rehabilitation (nine studies)^30^^,^^31^^,^^49^^,^^52^^,^^53^^,^^55^^,^^57^^,^^59^^,^^63^ and inpatient rehabilitation (six studies).^17^^,^^30^^,^^42^^,^^44^^,^^46^^,^^53^ Study designs included conceptual model proposals (12 studies),^30^^,^^32^^,^^38^^,^^49^^,^^52^^,^^55^^–^^60^^,^^63^ literature reviews (eight studies),^31^^,^^34^^,^^37^^,^^39^^,^^48^^,^^54^^,^^64^^,^^65^ qualitative articles, such as surveys, interviews, focus group discussions and Delphi methods (six studies),^40^^,^^41^^,^^45^^–^^47^^,^^51^ cohort studies (six studies),^32^^,^^35^^,^^36^^,^^42^^,^^44^^,^^53^ letters and practice pointers (four studies)^17^^,^^33^^,^^43^^,^^61^ and one RCT.^50^

**Table 1:** Studies included on rehabilitation care for post COVID-19 condition

Study and year	Country	Study design or type of publication	Population	Type of care models	Model of rehabilitation care setting
Ahmad et al., 2022^32^	United States	Cohort prospective and service model proposal	114 adults (mean age: 53 years); 76 (67%) females	NR	NR
Aiash et al., 2021^33^	Egypt	Correspondence	50 participants	NR	NR
Aiyegbusi et al., 2021^34^	United Kingdom	Literature review	NR	NR	NR
Albu et al., 2021^35^	Spain	Cohort prospective	40 participants (mean age: 52 years); 16 (40%) females	NR	Outpatient
Albu et al., 2021^36^	Spain	Cross-sectional	30 adults (mean age: 54 years)	NR	NR
Boutou et al., 2021^37^	Greece	Literature review	NR	NR	NR
Brigham et al., 2021^38^	United States	Service model proposal	NR	NR	Outpatient
Chaplin, 2021^39^	United Kingdom	Literature review	NR	NR	NR
Duncan et al., 2020^40^	United Kingdom (Scotland)	Survey	14 directors of allied health professions	NR	NR
Dundumalla et al., 2022^41^	United States	Survey	45 post-COVID clinics	NR	NR
Greenhalgh et al., 2020^17^	United Kingdom	Practice pointer	NR	Phase-adapted rehabilitation	Inpatient and outpatient
Gutenbrunner et al., 2021^42^	Germany	Development of a phase-adapted service model	NR	NR	Inpatient
Halpin et al., 2021^43^	United Kingdom	Response to letter	NA	NR	NR
Heightman et al., 2021^44^	United Kingdom	Cohort prospective	1325 adults (median age: 50 years); 748 (56%) females	One-stop shop	Inpatient and outpatient
Kingstone et al., 2020^45^	United Kingdom	Semistructured interviews	24 adults (age range: 20–68 years); 19 (79%) females	NR	NR
Ladds et al., 2020^46^	United Kingdom	Individual interviews and focus groups	114 participants, 32 doctors and 19 other health professionals (age range: 27–73 years); 80 (70%) females	One-stop-shop clinics	Inpatient and outpatient
Ladds et al., 2021^47^	United Kingdom	Individual interviews and focus groups	43 health-care professionals with long COVID (mean age: 40 years); 35 (81%) females	NR	NR
Lugo-Agudelo et al., 2021^48^	Colombia and Germany	Literature review	NR	NR	NR
Lutchmansingh et al., 2021^49^	United States	Service model proposal	NA	NR	Community
McGregor et al., 2021^50^	United Kingdom	RCT protocol	NR	NR	NR
Nurek et al., 2021^51^	United Kingdom	Delphi study	33 doctors from multiple specialties	NR	Outpatient
O’Brien et al., 2021^52^	Ireland	Service model proposal	NA	NR	Community
O’Sullivan et al., 2021^53^	United Kingdom	Cross-sectional	155 adults (median age: 39 years); 30 (18%) females	Three-tier model	Inpatient, outpatient and community
Parker et al., 2021^54^	United States	Literature review	NR	NR	NR
Parkin et al., 2021^55^	United Kingdom	Service model proposal	NA	NR	Outpatient and community
Pinto et al., 2020^56^	Italy	Service model proposal	NA	NR	NR
Postigo-Martin et al., 2021^30^	Spain	Surveillance model proposal	NA	Surveillance model for rehabilitation	Inpatient and community
Raza et al., 2021^57^	Pakistan	Service model proposal	NA	NR	Community
Santhosh et al., 2021^58^	United States	Service model proposal	NA	NR	NR
Shah et al., 2021^31^	United Kingdom	Literature review	NR	NR	Community
Sisó-Almirall et al., 2021^59^	Spain	Review and service model proposal	NR	NR	Outpatient and community
Sivan et al., 2020^60^	United Kingdom	Service model proposal	NA	NR	NR
Sivan & Taylor, 2020^61^	United Kingdom	Editorial	NA	NR	NR
Vanichkachorn et al., 2021^62^	United States	Service model report and cross-sectional	100 adults (mean age: 45 years); 68 (68%) Females	NR	Outpatient
Verduzco-Gutierrez et al., 2021^63^	United States	Service model proposal	NA	Post-COVID clinics, outpatient physiatry service	Outpatient and community
Wasilewski et al., 2022^64^	Canada	Scoping review	NR	NR	NR
Yan et al., 2021^65^	China	Literature review	NR	NR	NR

COVID-19: coronavirus disease 2019; NA: not applicable; NR: not reported; RCT: randomized controlled trial.

### Critical appraisal of articles

#### Topic 1

**Fig. 2:**
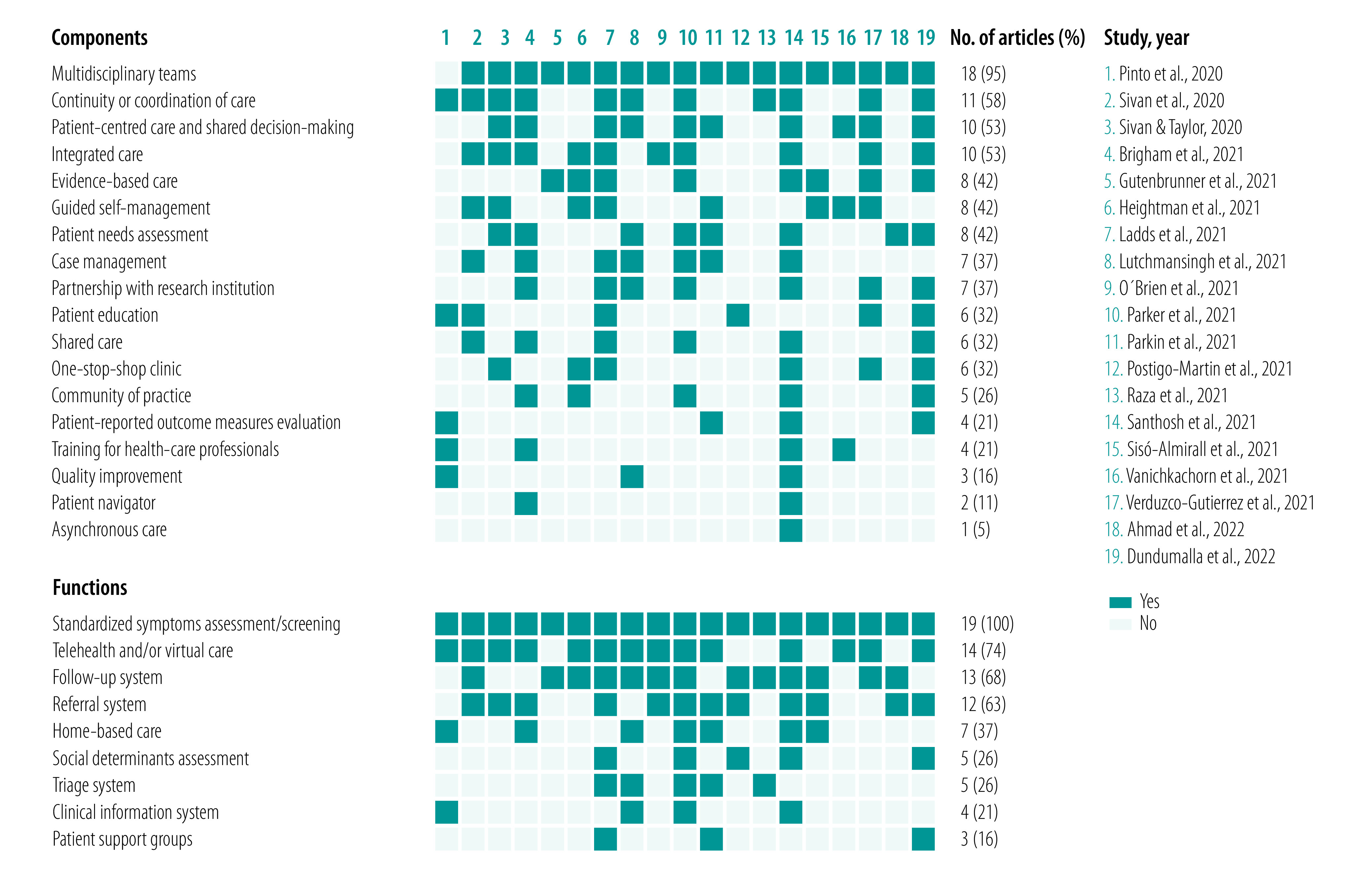
Proposed components and functions of rehabilitation care models for post COVID-19 condition

We identified 19 articles including information on components or functions (Fig. 2).^30^^,^^32^^,^^38^^,^^41^^,^^42^^,^^44^^,^^46^^,^^49^^,^^52^^,^^54^^–^^63^ A total of 18 components were described. The most common components were multidisciplinary rehabilitation teams (18 studies; 95%),^30^^,^^32^^,^^38^^,^^41^^,^^42^^,^^44^^,^^46^^,^^49^^,^^52^^,^^54^^,^^55^^,^^57^^–^^63^ continuity and coordination of care (11 studies; 58%),^38^^,^^41^^,^^46^^,^^49^^,^^54^^,^^56^^–^^58^^,^^60^^,^^61^^,^^63^ people-centred care and shared decision-making between clinicians and patients (10 studies; 53%),^38^^,^^41^^,^^46^^,^^49^^,^^54^^,^^55^^,^^58^^,^^61^^–^^63^ integrated care (10 studies; 53%),^38^^,^^41^^,^^44^^,^^46^^,^^52^^,^^54^^,^^58^^,^^60^^,^^61^^,^^63^ evidence-based care (eight studies; 42%),^41^^,^^42^^,^^44^^,^^46^^,^^54^^,^^58^^,^^59^^,^^63^ guided self-management (eight studies; 42%)^44^^,^^46^^,^^55^^,^^59^^–^^63^ and patient needs assessment (eight studies; 42%).^32^^,^^38^^,^^41^^,^^49^^,^^54^^,^^55^^,^^58^^,^^61^

COVID-19: coronavirus disease 2019.

A total of nine functions were described. The most common functions were standardized symptoms assessment and screening (19 studies; 100%),^30^^,^^32^^,^^38^^,^^41^^,^^42^^,^^44^^,^^46^^,^^49^^,^^52^^,^^54^^–^^63^ telehealth or virtual care (14 studies; 74%),^38^^,^^41^^,^^44^^,^^46^^,^^49^^,^^52^^,^^54^^–^^56^^,^^58^^,^^60^^–^^63^ follow-up system (13 studies; 68%),^30^^,^^32^^,^^42^^,^^44^^,^^46^^,^^49^^,^^52^^,^^54^^,^^57^^–^^60^^,^^63^ referral system (12 studies; 63%)^30^^,^^32^^,^^38^^,^^41^^,^^46^^,^^52^^,^^54^^,^^55^^,^^58^^–^^61^ and home-based care (seven studies; 37%).^38^^,^^49^^,^^54^^–^^56^^,^^58^^,^^59^

#### Topic 2

We identified three articles proposing symptoms that need further management.^17^^,^^30^^,^^31^ The articles did not directly address access to rehabilitation, but screening for conditions that could be addressed either with a different referral or during the rehabilitation process. One article proposed to screen for worsening breathlessness, the partial pressure of oxygen in the arterial blood below 96%, unexplained chest pain, new confusion or focal weakness.^17^ One article proposed to screen using a cardiopulmonary exercise test.^30^ One article proposed to refer people with post COVID-19 condition to the relevant acute services if they have signs or symptoms including orthostatic intolerance (e.g. palpitation or dizziness on standing), oxygen desaturation on exercise, signs of severe lung disease or cardiac chest pain.^31^

#### Topic 3

We identified 16 articles reporting on rehabilitation referral principles.^17^^,^^30^^–^^32^^,^^35^^,^^37^^–^^40^^,^^44^^,^^54^^,^^55^^,^^58^^,^^60^^,^^62^^,^^63^ Fifteen articles provided information on criteria for referral to rehabilitation.^17^^,^^30^^–^^32^^,^^35^^,^^37^^–^^39^^,^^44^^,^^54^^,^^55^^,^^58^^,^^60^^,^^62^^,^^63^ The content analysis of each article is available in the data repository.^25^ Eleven articles reported that the main criteria for referral into rehabilitation were based on any new or persistent symptoms from COVID-19. Six articles mentioned the importance of ruling out other diagnoses or urgent medical conditions and possible reinfection before referral into rehabilitation. Three articles additionally emphasized that symptoms should have an impact on functioning and quality of life to be eligible for referral. Two articles proposed the use of a standardized tool including referral criteria based on expert consensus (i.e. COVID-19 Yorkshire Rehabilitation Scale).^66^ No article mentioned severity as a criterion for rehabilitation. All included articles valued a personalized testing and assessment procedure with no information of a core assessment procedure.

Eleven articles provided information on timing for referral to rehabilitation,^17^^,^^32^^,^^35^^,^^37^^,^^38^^,^^40^^,^^55^^,^^58^^,^^60^^,^^62^^,^^63^ however, without considering the duration of 3 months from the onset of COVID-19. The content analysis of each article is available in the data repository.^25^ Eight studies recommended immediate referral following hospitalization. For patients in the community, the timing for referral occurs following assessment from family physicians, nurses or other health-care workers, usually without strict temporal criteria. Three articles highlighted a self-referral and direct access process for timing of rehabilitation. Four studies recommended a wait-and-see approach or self-management approach of at least 6 weeks following symptoms to observe possible natural recovery before referral to rehabilitation.

#### Topic 4

We identified 24 articles reporting on settings for rehabilitation services delivery (Table 2).^17^^,^^31^^,^^32^^,^^34^^,^^35^^,^^38^^–^^42^^,^^44^^,^^45^^,^^47^^,^^48^^,^^50^^,^^52^^,^^54^^–^^56^^,^^60^^–^^63^^,^^65^ The main delivery modes were face-to-face and virtual delivery. Fourteen articles proposed type of rehabilitation care organization, such as hospital settings, community, one-stop-shop clinics, outpatient physiatry clinics or integrated into primary care. Five articles proposed support mechanisms to facilitate delivery of rehabilitation services, such as follow-up appointments, general practitioner's support, prolonged monitoring and use of technology platforms. Concerning integrated or parallel rehabilitation services, some articles suggested developing parallel services (e.g. specialty clinics), while other articles proposed integrated care within health systems’ current services. Some articles proposed a fixed 8-week or 12-week length of programme, while others proposed an individualized approach.

**Table 2:** Themes identified concerning rehabilitation service delivery settings for post COVID-19 condition

Classification and themes	No. (%) of studies (*n* = 24)
**Rehabilitation delivery mode**	
Face-to-face^35^^,^^40^^,^^41^^,^^45^^,^^47^^,^^50^^,^^54^^,^^56^^,^^61^^–^^63^^,^^65^	12 (50)
Virtual^17^^,^^40^^,^^41^^,^^50^^,^^54^^–^^56^^,^^61^^–^^63^^,^^65^	11 (44)
Group therapy in person^35^	1 (4)
Virtual group therapy^55^	1 (4)
Self-management^17^	1 (4)
Not reported^31^^,^^32^^,^^34^^,^^39^^,^^42^^,^^44^^,^^48^^,^^52^^,^^60^	9 (38)
**Type of rehabilitation care organization**	
Hospital-based^32^^,^^38^^,^^52^^,^^62^	4 (17)
Community-based^52^^,^^60^	2 (8)
One-stop-shop clinic^a,^^41^^,^^44^^,^^47^^,^^61^	4 (17)
Outpatient physiatry clinic^63^	1 (4)
Primary care collaboration^60^	1 (4)
Locally-based^52^	1 (4)
Nationally-based^55^	1 (4)
Not implemented^17^^,^^56^^,^^65^	3 (13)
Not reported^35^^,^^40^^,^^45^^,^^50^^,^^54^^,^^60^	6 (25)
**Support mechanisms to facilitate delivery of rehabilitation services**	
Follow-up appointment^32^^,^^47^^,^^54^	3 (13)
General practitioners supporting the patient^45^^,^^47^	2 (8)
Prolonged monitoring^32^	1 (4)
Provided recommendation to general practitioners at discharge^32^	1 (4)
Best transition of care^32^	1 (4)
Integration of other health professional^47^	1 (4)
Connection between health professionals and patients via an internet-based platform^56^	1 (4)
Not reported^17^^,^^31^^,^^34^^,^^35^^,^^38^^–^^42^^,^^44^^,^^48^^,^^50^^,^^52^^,^^55^^,^^61^^–^^63^^,^^65^	18 (75)
**Integrated or parallel rehabilitation service**	
Parallel rehabilitation service^17^^,^^35^^,^^38^^,^^44^^,^^52^^,^^55^^,^^60^^,^^65^	8 (33)
Integrated rehabilitation service^32^^,^^47^^,^^55^^,^^56^^,^^60^^–^^62^	7 (29)
Developing referral criteria^60^	1 (4)
Not reported^31^^,^^34^^,^^39^^–^^42^^,^^45^^,^^48^^,^^50^^,^^54^^,^^63^	11 (46)
**Length of programme**	
Individually estimated^32^^,^^40^	2 (8)
8 weeks^35^^,^^50^	2 (8)
12 weeks^63^	1 (4)
Not reported^17^^,^^31^^,^^34^^,^^38^^,^^39^^,^^41^^,^^42^^,^^44^^,^^45^^,^^47^^,^^48^^,^^52^^,^^54^^–^^56^^,^^60^^–^^62^^,^^65^	19 (79)

COVID-19: coronavirus disease 2019.

^a^ One-stop-shop clinic is a clinic where the patient sees all care team members and subspecialists in the same clinical visit.

#### Topic 5

**Fig. 3:**
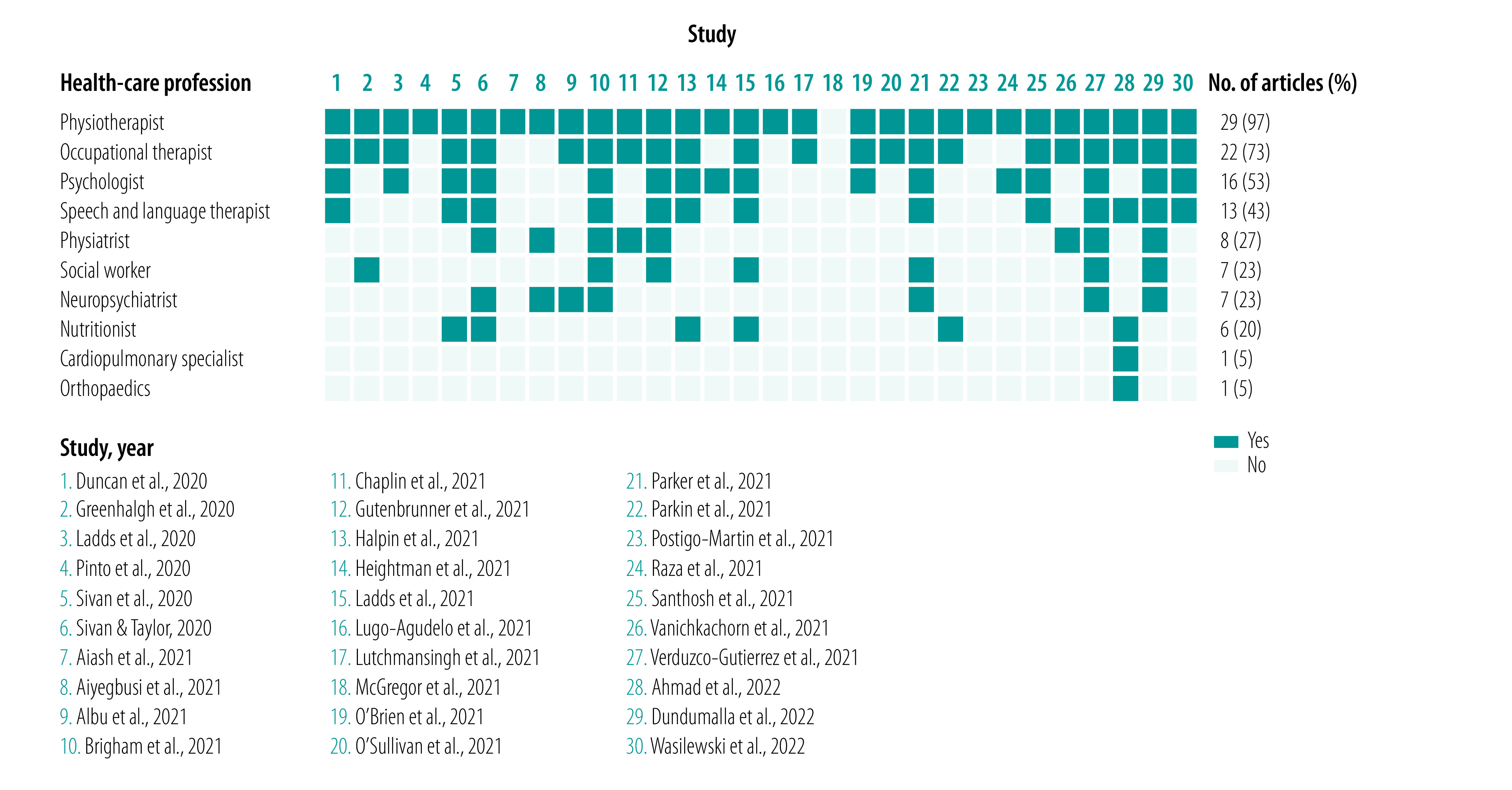
Health-care workers providing rehabilitation interventions for post COVID-19 condition

We identified 30 articles proposing health-care professionals to deliver rehabilitation services for post COVID-19 condition (Fig. 3).^17^^,^^30^^,^^32^^–^^35^^,^^38^^–^^44^^,^^46^^–^^50^^,^^52^^–^^58^^,^^60^^–^^64^ Ten different professions were proposed. The most common professionals were physiotherapists (29 studies; 97%),^17^^,^^30^^,^^32^^–^^35^^,^^38^^–^^44^^,^^46^^–^^49^^,^^52^^–^^58^^,^^60^^–^^64^ occupational therapists (22 studies; 73%),^17^^,^^32^^,^^35^^,^^38^^–^^43^^,^^46^^,^^47^^,^^49^^,^^52^^–^^55^^,^^58^^,^^60^^–^^64^ psychologists (16 studies; 53%),^38^^,^^40^^–^^44^^,^^46^^,^^47^^,^^52^^,^^54^^,^^57^^,^^58^^,^^60^^,^^61^^,^^63^^,^^64^ speech and language therapists (13 studies; 43%),^32^^,^^38^^,^^40^^–^^43^^,^^47^^,^^54^^,^^58^^,^^60^^,^^61^^,^^63^^,^^64^ physiatrists (eight studies; 27%),^34^^,^^38^^,^^39^^,^^41^^,^^42^^,^^60^^,^^62^^,^^63^ social workers (seven studies; 23%),^17^^,^^38^^,^^41^^,^^42^^,^^47^^,^^54^^,^^63^ neuropsychiatrists (seven studies; 23%)^34^^,^^35^^,^^38^^,^^41^^,^^54^^,^^60^^,^^63^ and dieticians or nutritionists (six studies; 20%).^32^^,^^43^^,^^47^^,^^55^^,^^60^^,^^61^ We found no information concerning the type of competencies and skills nor the number of years of clinical experience required for working with post COVID-19 condition.

COVID-19: coronavirus disease 2019.

### Concept map

**Fig. 4:**
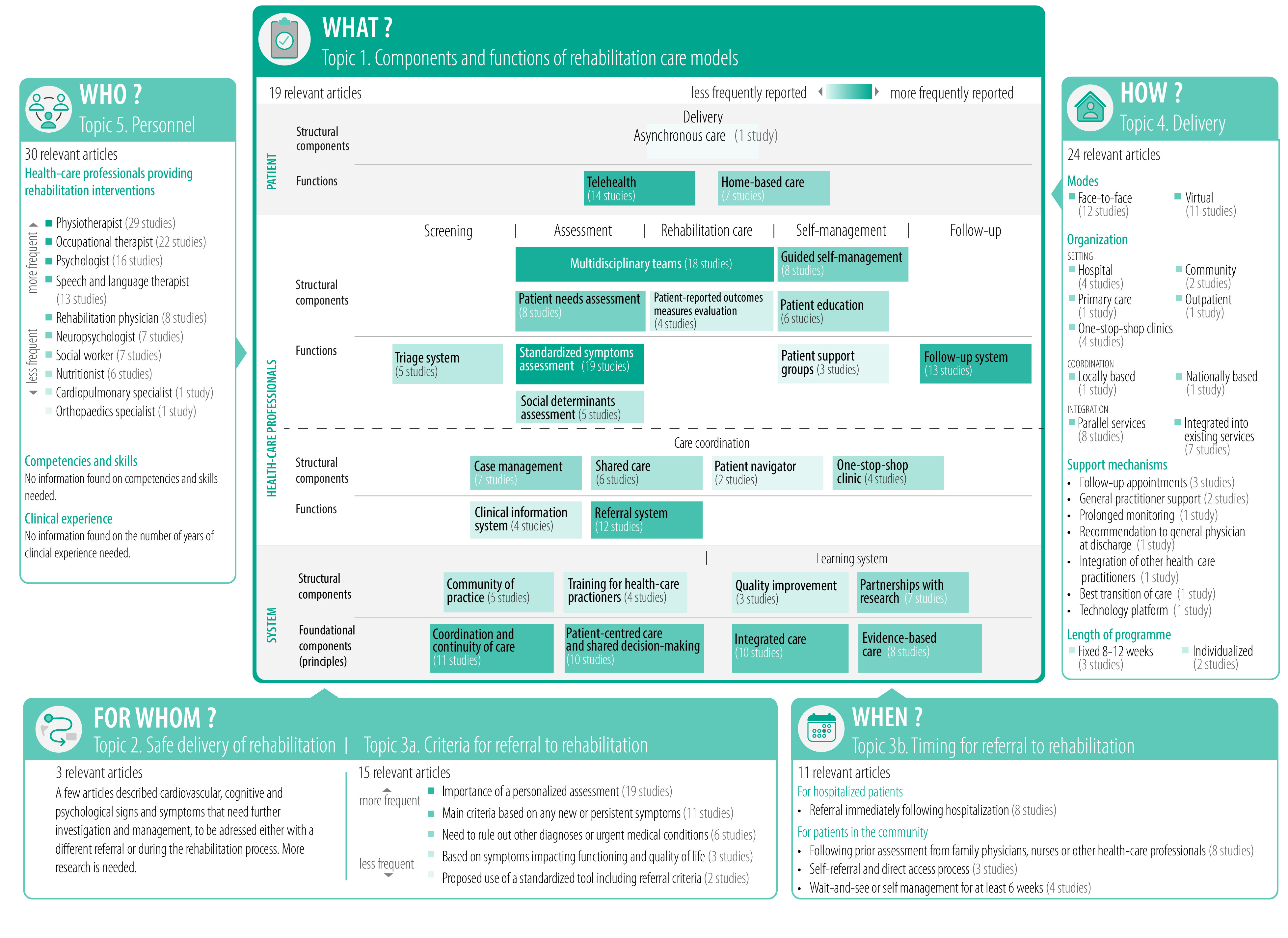
Concept map for the design of a rehabilitation care model for post COVID-19 condition

Fig. 4 presents a concept map to guide decision-makers in designing sustainable rehabilitation care models for post COVID-19 condition. 

COVID-19: coronavirus disease 2019.

## Discussion

Here we provide current evidence on health system, providers and patients’ characteristics for care models for post COVID-19 condition. Considering that the evidence retrieved is conceptual, expert based, with no high-quality trials, below we guide decision-makers on how to locally adapt rehabilitation services for post COVID-19 condition within their health systems and we provide key policy messages for decision-makers (Box 2). We also highlight the evidence gaps for researchers to answer.

Box 2 **Key policy messages on care models for post COVID-19 condition**

1. Rehabilitation care for post COVID-19 condition should not be limited to one specific approach or profession; we suggest a multilevel and multiprofessional care model.
2. Due to the complexity of post COVID-19 condition, decision-makers should leverage all available strengths of their own health system, learn and adapt from experience with other conditions to provide appropriate rehabilitation.
3. Financing for rehabilitation of people with post COVID-19 condition should include funding programmes and research using standardized measurements that target contextualized and prioritized health system needs for optimal rehabilitation outcomes.
4. Policy-making for rehabilitation of people with post COVID-19 condition should include guidance for researchers and clinicians to develop and adopt appropriate mechanisms to increase patient safety.

COVID-19: coronavirus disease 2019.

Decision-makers need to consider that current care model proposals worldwide were developed based on expert opinions and may be linked to conflict of interest and biased perspectives (e.g. country of origin, professions). For example, most included articles come from high- to upper-middle-income countries. The proposed components and functions will probably face applicability issues within different health systems. For example, decision-makers in the United Kingdom were the first to propose a highly financed post-COVID clinics network organized at the national level. Although inspiring, the applicability to other health systems is unknown because of funding sustainability. Hence decision-makers need to locally adapt rehabilitation services for post COVID-19 condition within their health systems.

While a multidisciplinary team approach appeared as the most prevalent component of rehabilitation care models, articles reported only a few professions as key providers. Pivotal rehabilitation professions may be underrepresented in studies such as physiatrists, psychologists, speech and language therapists or dieticians. This potential underrepresentation in evidence also extends to the specifics of patients assessed within rehabilitation care models. The focus was mostly on a single body structure or function, rarely considering multiple impairments and limitations in functioning. Most articles reporting on patients’ outcomes had a small sample size of previously hospitalized or non-hospitalized patients. We cannot expect that current components and functions of the care model will be applicable to all patients within the heterogeneous presentations of post COVID-19 condition.

Concepts of care models appear to spur from the current understanding of rehabilitation.^67^ However, rehabilitation deals with complex interrelations of comorbidities with different courses (e.g. acute onset, progressive, episodic or relapsing remitting) for many conditions. Yet not one care model may be transferable to post COVID-19 condition. To create effective care models, we must disentangle components and functions that are specific to post COVID-19 condition, while leveraging effective practices used for other disabling conditions. Described components such as multiprofessional rehabilitation teams, continuity or coordination of care, or people-centred care, highlight the importance of interdisciplinary work and the involvement of patients regarding their preferred rehabilitation services and outcomes. Other components address education of patients and self-management as integral parts of case management. The reported functions suggest a care model supported with a standardized monitoring system which allows referrals based on patient needs and an option of home-based care that may be delivered with telerehabilitation services. We argue that these components should be standard practice for rehabilitation of conditions with complex and chronic rehabilitation needs. 

A challenge in designing a care model for post COVID-19 condition is that it needs to consider people who gradually recover, people who experience episodic disability^68^ and people who may be facing a permanent disability.^69^ We can hypothesize that clinical experience in other chronic conditions, such as neurological diseases, cancer or cardiovascular diseases could yield superior patient outcomes by adapting indirect evidence for post COVID-19 condition patients.

Further research is needed as we found limited information to identify the optimal rehabilitation service delivery setting. Decision-makers could consider a hybrid delivery mode including face-to-face or virtual mode, but the evidence concerning its safety, effectiveness or non-inferiority to traditional delivery in an outpatient setting is still lacking. Integrating rehabilitation services at many different levels of health care from primary care to hospital-based care is probably feasible, but trials of rehabilitation care models and pathways are still lacking for post COVID-19 condition. We could not determine the ideal length of a rehabilitation programme. Limited information does not allows us to fully identify a core multidisciplinary team of health-care workers providing rehabilitation interventions for post COVID-19 condition. The nature of their professional implication is yet to be determined (e.g. number of sessions, intensity, exposure and interventions). Our concept map could help researchers to develop care model interventions to assess their impact and cost–effectiveness in RCTs.

We observed a scarcity of information concerning safe delivery of rehabilitation with the assessment of signs and symptoms that prevent the admission of a patient to rehabilitation, at least temporarily and when not medically managed. We found only a few articles describing signs and symptoms of the cardiovascular, cognitive and psychological domains that need further investigation and management without specifically mentioning the need for their management to ensure safe rehabilitation. Clinicians have already identified contraindications for physical activity interventions in post COVID-19 condition, such as cardiac impairment following COVID-19 and post-exertional symptom exacerbation.^70^ Authors of all articles recommended personalized assessments, which included detailed history of the disease, clinical examination, activity tolerance (e.g. physical or cognitive) and impact of symptoms on functioning, but no article considered standardized assessment of conditions that prevent safe delivery of rehabilitation. This lack of information may be driven by underreporting of potential negative consequences such as adverse events and harms in rehabilitation studies. Suboptimal reporting of harms and adverse events is an ongoing issue in randomized trials and rehabilitation studies.^71^ Future research on rehabilitation for post COVID-19 condition should identify prevalence of harms and adverse events during and after rehabilitation to guide standardized safety netting within care models.

As the pandemic evolves into endemicity, more people will develop post COVID-19 condition each year for the foreseeable future, even with vaccine protection.^72^ We have gathered four key policy messages for decision-makers and researchers for developing and improving rehabilitation care for people living with post COVID-19 conditions (Box 2). We suggest a multilevel and multiprofessional model, where decision-makers should leverage all available strengths and experiences of their own health system to provide rehabilitation services by funding programmes and research that aim for optimal rehabilitation outcomes. We also suggest providing guidance for researchers and clinicians to develop and adopt appropriate mechanisms to increase patient safety. Decision-makers can use our concept map, keeping in mind the current state of evidence, to design potentially effective, locally adapted and sustainable rehabilitation care models for post COVID-19 condition.
